# Insulin resistance’s role in mediating physical activity’s impact on beta amyloid accumulation and tau phosphorylation: A scoping review

**DOI:** 10.1002/alz.090557

**Published:** 2025-01-09

**Authors:** Michael G Slee, Joanne Scotney, Stephanie R Rainey‐Smith, Kirk I. Erickson, Hamid R. Sohrabi, Belinda M. Brown

**Affiliations:** ^1^ Centre for Healthy Ageing, Murdoch University, Murdoch, Western Australia Australia; ^2^ Murdoch University, Perth, Western Australia Australia; ^3^ School of Psychological Science, University of Western Australia, Crawley, Western Australia Australia; ^4^ Centre of Excellence for Alzheimer’s Disease Research and Care, School of Medical and Health Sciences, Edith Cowan University, Joondalup, Western Australia Australia; ^5^ Australian Alzheimer’s Research Foundation, Nedlands, Western Australia Australia; ^6^ AdventHealth Research Institute, Neuroscience, Orlando, FL USA; ^7^ University of Pittsburgh, Pittsburgh, PA USA; ^8^ Department of Biomedical Sciences, Macquarie University, Sydney, NSW Australia; ^9^ Centre for Healthy Ageing, Murdoch University, Murdoch, Perth, Western Australia Australia; ^10^ Edith Cowan University, Perth, Western Australia Australia; ^11^ Alzheimer’s Research Australia, Perth, Western Australia Australia

## Abstract

**Background:**

Type 2 diabetes (T2D) and Alzheimer’s disease (AD) are amongst the top 10 leading causes of death worldwide. T2D is associated with increased AD risk and brain beta amyloid (Aβ) burden, suggesting underlying mechanistic relationship between AD and T2D. Insulin signalling pathways, which can regulate the accumulation of Aβ and phosphorylation of tau in the brain, is a possible underlying mechanism of the T2D‐AD relationship. Insulin resistance, reduced responsiveness to insulin and T2D hallmark, has been found to impair insulin signalling pathway function. Animal studies show exercise reduces brain levels of Aβ. Whilst empirical evidence from human studies linking exercise to brain Aβ is somewhat limited, some studies have reported physical activity is associated with lower brain Aβ. Exercise has well established links to reductions in insulin resistance; thus, as physical activity can impact both insulin resistance and AD biomarkers, it is reasonable to hypothesise that a mediating relationship may exist. The objective of this review was to identify what evidence exists that examines the association between insulin, physical activity, and Aβ/tau in animals and human studies. Particularly, what is known about the potential mediating role insulin resistance has on the physical activity ‐ Aβ/tau relationship.

**Method:**

A systematic search was performed in Cochrane library, PsycINFO, PubMed and World of Science to identify suitable publications. The search identified 343 articles with 21 articles meeting the full inclusion criteria.

**Result:**

Most animal studies showed that exercise could simultaneously reduce insulin resistance and AD pathology. Limited human studies were identified and only a few found both reduced insulin resistance and Aβ/tau associated with higher physical activity. One study provided evidence that insulin resistance may mediate the physical activity ‐ Aβ relationship, however this was not derived through standard mediation analysis.

**Conclusion:**

Exercise can simultaneously impact insulin resistance and AD pathology in animal models, suggesting similar underlying mechanisms. Results in human models are limited however, and no robust evaluation of insulin resistance’s mediating role in the physical activity ‐ Aβ/tau relationship exists. Future focus on mediation impact should be investigated to enhance our understanding of physical activity’s role in reducing AD risk.

